# Correlation analysis of human upper arm parameters to oscillometric signal in automatic blood pressure measurement

**DOI:** 10.1038/s41598-022-24264-9

**Published:** 2022-11-17

**Authors:** Bomi Lee, Jae-Hak Jeong, Junki Hong, Yong-Hwa Park

**Affiliations:** Department of Mechanical Engineering, Korea Advanced Institute of Science and Technology, Daejeon, 34141 Republic of Korea

**Keywords:** Health care, Biomedical engineering, Mechanical engineering

## Abstract

Cardiovascular diseases are the leading cause of global deaths, making cardiovascular health monitoring important. Measuring blood pressure using an automatic sphygmomanometer is the most widely used method to monitor cardiovascular health due to its accessibility, convenience, and strong correlation with cardiovascular diseases. In this work, in order to estimate brachial artery diameter, stiffness, or thickness using an automatic sphygmomanometer, the correlation between upper arm parameters and the oscillometric signal was intensively investigated through analytical, numerical, and experimental approaches. The parametric studies commonly revealed that the inner radius of the brachial artery is the most influential parameter in determining the amplitude of the oscillometric signal. The experimental results of using a cardiovascular simulator (a *virtual patient*) combined with upper arm phantoms with various inner radii of the brachial artery showed a 6.5% change in the oscillometric signal amplitude with a 10% artery radius variation. It was concluded that the oscillometric signal can be used to evaluate brachial artery diameter. Based on the clinical relationship between brachial artery diameter and cardiovascular risk factors such as hypertension, diabetes, and obesity, this study showed and verified a novel method to monitor brachial artery diameter and hence, cardiovascular risks while measuring blood pressure.

## Introduction

Cardiovascular diseases (CVDs) are the number one cause of global death. In 2019, nearly 18 million people died from CVDs, which accounts for almost one-third of the total death^1^. The major causes of such CVDs are arteriosclerosis and atherosclerosis^2,3^ where the vessels become narrow and stiff, hindering blood flow. Such changes in the vessels are accompanied by an increase in blood pressure (BP), leading to hypertension, which is a representative symptom and factor of CVDs^4^. Since such degenerative changes in vessels are progressive and lead to severe diseases, monitoring vessel health is essential for the early diagnosis and treatment of CVDs.

So far, monitoring the vessel health has been done by checking the stiffness and occlusion of vessels which are examined in the hospital with dedicated medical devices. A typical method of measuring arterial stiffness is pulse wave velocity (PWV) by measuring the pulse at two different positions—for instance, carotid-femoral^5,6^, brachial-ankle^7,8^, or finger-toe^9^—and calculating the transit time the pulse took to propagate. Another method to estimate vessel stiffness includes the augmentation index (AIx), which is calculated from the blood pressure waveform obtained invasively with a catheter or non-invasively with tonometry^10–12^. Angiography is a well-known examination to check the vessel diameter and hence, the level of occlusion^13^. However, as those methods need clinicians and special medical devices, they are not proper for a regular, frequent check-up of vessel health from the user aspect. Hence, recent studies focus on extracting vessel-related information from more accessible devices or signals. One of the most widely studied signals is photoplethysmography (PPG)^14–21^, where the contour of the pulse wave can be obtained from the fingertip or wrist. Even though PPG signals can be easily recorded through wearable devices, the signals are vulnerable to small movement, making use of the signals for in-depth evaluation inappropriate.

**Figure 1:**
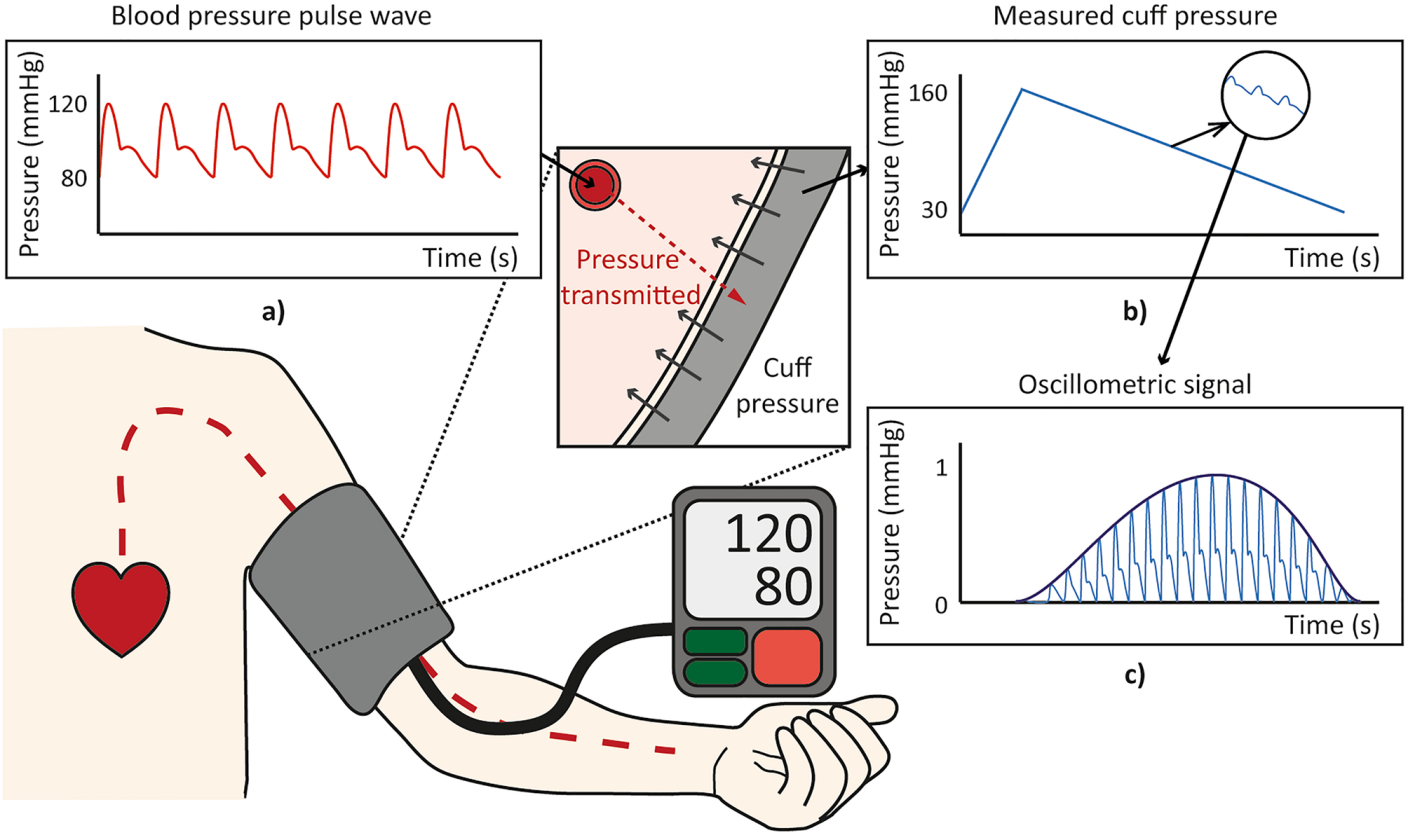
An oscillometry-based automatic sphygmomanometer. Blood pressure pulses are propagated from the heart to the brachial artery, and the pressure is transmitted to the cuff of the device through upper arm parameters. The cuff pressure and corresponding oscillometric signal are recorded by the automatic sphygmomanometer. (**a**) Blood pressure pulse wave inside brachial artery, (**b**) Cuff pressure measured by automatic sphygmomanometer, (**c**) Oscillometric signal, which records cuff pressure oscillation.

In contrast, BP, which is one of the simplest but most powerful indicators of blood vessel health, can be easily measured using automatic sphygmomanometer without clinician assistance. Indeed, due to the strongest evidence for causation of CVDs^4^, measuring BP on a regular basis is recommended for those with a high CVD risk. Moreover, the oscillometric signal obtained during the BP measurement, as shown in Fig. 1, is relatively stable as the measurement is taken at resting condition. In order to take advantage of the simple, stable, and already widely used automatic sphygmomanometer, the possibility of extracting cardiovascular health parameters such as brachial artery diameter, elasticity, and thickness from the oscillometric signal is investigated in this work.

Previous studies regarding oscillometric signals have focused on either the modeling of the blood pressure measurement process^22–26^ or the simple hardware simulator that can calibrate an automatic sphygmomanometer^27–30^ to improve automatic sphygmomanometer accuracy. Some research has used the oscillometric signal to investigate cardiovascular-related parameters such as vascular endothelial function^2^. However, as far as we know, no previous work has investigated oscillometric signals with use of a cardiovascular simulator and phantoms with the aim of analyzing the correlation between oscillometric signals and cardiovascular-related parameters. Thus, with the ultimate goal of developing an oscillometric-based sphygmomanometer that can monitor blood vessel health, our work utilized analytical, numerical modeling, and experimental approaches, and examined the correlation between oscillometric signals and upper arm parameters. Analytical modeling with a simplified, axi-symmetric upper arm model was used to reveal the underlying dominant physics in the pressure transmission from the brachial artery to the air cuff. Finite element method (FEM)-based analyses with a simplified upper arm model and a magnetic resonance imaging (MRI)-based, complicated model were additionally investigated to undertake more detailed parametric studies that could not be considered in the analytical model. An experiment with a cardiovascular simulator and upper arm phantoms fabricated using 3D-printing molds that mimicked the human circulatory system and upper arm was conducted as mutual verification of the parametric study results from the MRI-based FEM and the experiment.

This work had two main objectives. First, with the understanding of the oscillometric signal and with the upper arm composition, parametric studies between the upper arm parameters and the oscillometric signal were carried out to identify the parameters that affected the oscillometric signal the most. The result was verified by physical experiment with the phantoms and cardiovascular simulator. Second, based on the results from the parametric studies verified by experiment, but utilizing the results in a reverse way, it was examined whether vessel diameter, elasticity, and stiffness could be extracted from the oscillometric signal. The results confirmed a high correlation between the oscillometric signal amplitude and the inner radius of the brachial artery, demonstrating that it is possible to extract brachial artery diameter from oscillometric signals.

## Methods

### Modeling with analytic and numerical approaches

**Figure 2:**
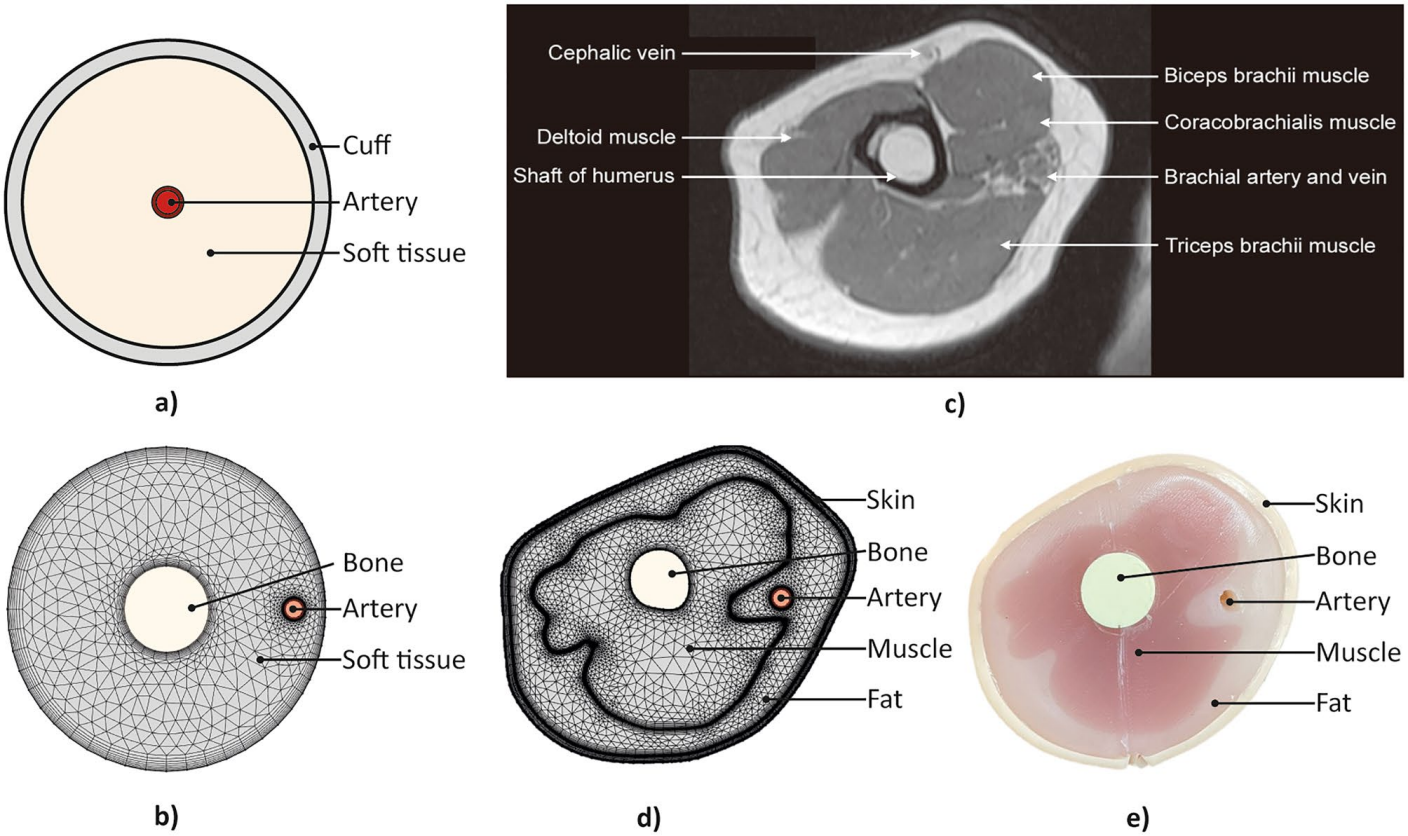
Models and phantom configuration for parametric study and experiment. (**a**) Analytical model based on pressure propagation through concentric components, (**b**) Simple FEM model based on the simplified human arm anatomy, (**c**) Actual MRI image of cross-section of human upper arm, (**d**) MRI-based FEM model from the MRI image in (**c**), (**e**) Cross-sectional view of fabricated phantom based on the MRI image in (**c**).

In order to identify the effect of upper arm parameters on pressure transmission, the correlation between the upper arm parameters and the oscillometric signal was analyzed via parametric studies with various 2D models (Fig. 2), focusing on pressure transmission in a radial direction. The upper arm parameters included geometrical and mechanical properties of the brachial artery and soft tissue (muscle, fat, and skin), which were subdivided depending on the model complexity. The parametric studies were conducted with three progressive models with increasing complexity, as shown in Fig. 2a, b, and d. For each modeling and analysis, brachial blood pressure, ranging from 80 to 120 mmHg with heart beat of 70 bpm, was considered as the pressure exerting on the inner wall of the brachial artery, rather than considering the blood flow through it. Also, the cuff pressure, inflating from 0 to 160 mmHg for 3 s and deflating to 0 mmHg during 27 s, was set as a boundary condition at the arm surface to model the slowly varying control pressure of the cuff surrounding the upper arm. The non-linear behavior of the blood vessel to the different transmural pressure (i.e., internal pressure minus external pressure of the blood vessel) was implemented to reflect pressure-varying compliance of the blood vessel, which results in the known bell shape of the oscillometric signal. The non-linear behavior of the blood vessel can be expressed using the arterial pressure–volume model^31^ as

1 $$V = \left\{ {\begin{array}{*{20}l} {V_{0} e^{{\left( {C_{m} /V_{0} } \right)P_{t} }} } \\ {V_{m} - \left( {V_{m} - V_{0} } \right)e^{{ - C_{m} /\left( {V_{m} - V_{0} } \right)P_{t} }} } \\ \end{array} } \right.\begin{array}{*{20}c} {{\text{ for }}P_{t} \le 0} \\ {{\text{ for }}P_{t} > 0} \\ \end{array}$$

where $P_{t}$ is the transmural pressure, $V$ is the arterial volume at $P_{t}$, $V_{0}$ is the arterial volume at zero $P_{t}$, $V_{m}$ is the maximum arterial volume, and $C_{m}$ is the maximum vessel compliance. The change of the inner radius of the brachial artery against the transmural pressure is calculated based on the volume-pressure relationship in Eq. (1), and the calculated inner radius is used at each transmural pressure point depending on the brachial blood pressure and the cuff pressure set earlier.

#### Analytical model

Analytical modeling and analysis were carried out to understand the underlying dominant behavior of the pressure transmission from the brachial artery to the cuff. For calculational simplicity, the model was simplified, as shown in Fig. 2a, such that the blood vessel was placed at the center of the arm, surrounded by soft tissue and the air-filled cuff. With the simplification, the blood vessel, arm, and cuff could be modeled as concentric circular cylinders. It was assumed that the process was quasi-static (slowly time-varying enough to be in a statically equilibrium state to the blood pressure input), which can be reasonably accepted since the resonant frequency of an arm in radial motion, normally larger than 10 Hz, is sufficiently larger than the dominant frequency of the blood pressure profile, which is about a few Hertz. All the components were assumed isotropic and the deformations of the components were assumed to be within the linear range. The air inside the cuff was assumed to be in the isentropic process^22^. The thicknesses of the inner and outer cuff layers were assumed to be negligible, and the outer cuff layer was assumed to be fixed in position while the inner layer was movable along the outmost part of the arm soft tissue, causing the pressure oscillation of the air in the cuff. The blood vessel and soft tissue layers were modeled as pressurized thick-wall cylinders. Finally, the radial directional pressure and the displacement for each cylindrical layer can be expressed as the following matrix–vector form based on the pressurized thick-wall cylinder law^32^

2 $$\left[ {\begin{array}{*{20}c} \sigma \\ {\Delta r} \\ \end{array} } \right] = {\varvec{T}}_{{\varvec{X}}} \left[ {\begin{array}{*{20}c} {p_{i} } \\ {p_{o} } \\ \end{array} } \right]$$

where $\sigma$ is radial stress, $\Delta r$ is radial displacement, $p_{i}$ is inner pressure, $p_{o}$ is outer pressure, and ${\varvec{T}}_{{\varvec{X}}}$ is a transfer matrix determined by the mechanical properties and geometries of the corresponding cylindrical layer ${\varvec{X}}$, as shown in

3 $${\varvec{T}}_{{\varvec{X}}} = \left[ {\begin{array}{*{20}c} {\frac{{r_{i}^{2} }}{{r_{o}^{2} - r_{i}^{2} }}\left( {1 - \frac{{r_{o}^{2} }}{{r^{2} }}} \right)} & { - \frac{{r_{o}^{2} }}{{r_{o}^{2} - r_{i}^{2} }}\left( {1 - \frac{{r_{i}^{2} }}{{r^{2} }}} \right)} \\ {\frac{{r_{i}^{2} }}{{r_{o}^{2} - r_{i}^{2} }}\left( {\frac{{\left( {1 - 2\nu } \right)\left( {2 + \nu } \right)r + \left( {1 + \nu } \right)\frac{{r_{o}^{2} }}{r}}}{E}} \right)} & { - \frac{{r_{o}^{2} }}{{r_{o}^{2} - r_{i}^{2} }}\left( {\frac{{\left( {1 - 2\nu } \right)\left( {2 + \nu } \right)r + \left( {1 + \nu } \right)\frac{{r_{i}^{2} }}{r}}}{E}} \right)} \\ \end{array} } \right]$$

where $r_{i}$ is inner radius, $r_{o}$ is outer radius, $\nu$ is Poisson’s ratio, and $E$ is elastic modulus of the corresponding cylindrical layer ***X***. From the geometric compatibility and force equilibrium at the interface of adjacent cylindrical layers such that the outer displacement and pressure of the blood vessel and the inner displacement and pressure of the soft tissue are equal, respectively, the radial displacement transmitted to the inner cuff layer could be calculated. Then, the volume change of the cuff induced by the change in displacement of the inner cuff layer can be calculated and then utilized to obtain the pressure change inside the cuff, based on the equation representing the isentropic process in the cuff as follows:

4 $$P_{cuff} V_{cuff}^{k} = {\text{constant}}$$

where $P_{cuff}$ is pressure inside the cuff, $V_{cuff}$ is volume of the cuff, and $k = 1.4$ for the isentropic process. Since the oscillometric signal indicates the oscillation of the air pressure in the cuff with the slowly varying inflation/deflation pressure component removed^33^ (as illustrated in Fig. 1c), this inflation/deflation trend of $V_{cuff}$ was removed by subtracting the trend line shown in Fig. 1b from the obtained pressure $P_{cuff}$, resulting in the oscillometric signal shown in Fig. 1c.

For the blood vessel, the inner and outer radii were set to 2.25 and 2.75 mm, respectively, and the Poisson’s ratio and elastic modulus were set to 0.45 and 450 kPa, respectively^23,30^. For the soft tissue, the inner and outer radii were set to 2.75 mm and 45 mm, respectively, and the Poisson’s ratio and elastic modulus were set to 0.45 and 47.5 kPa, respectively^34^. With the analytical model, the artery radius, artery elasticity, artery thickness, and the soft tissue elasticity were varied for the parametric study. The baseline values were 2.25 mm, 450 kPa, 0.50 mm, and 47.5 kPa, respectively, and the values were decreased and increased by 10% to examine the corresponding changes in the oscillometric signal.

#### Finite element method (FEM) model

In order to reflect a more realistic structure of the upper arm, which was not possible with the analytical model, numerical FEM models were built for the parametric study with COMSOL Multiphysics^®^ software. A simple FEM model considers the eccentric position of the brachial artery and the existence of the bone, as shown in Fig. 2b. In addition, an MRI-based FEM model reflects the cross-sectional MRI image of an actual human upper arm^35^ (Fig. 2c) to construct the model, subdividing the soft tissue into muscle, fat, and skin, as shown in Fig. 2d. In order to analyze the effect of the parameter variations on the oscillometric signal, the surface oscillation at the outmost part was calculated using the FEM, and the air volume change in the cuff was obtained in the same way as in the analytical modeling, using Eq. (4). For the finite elements, triangular elements were used for the soft tissue (Fig. 2b) and the subdivided components (Fig. 2d), and quadratic elements were used for the boundary between each component.

For the simple FEM model shown in Fig. 2b, the bone, soft tissue, and artery had radii of 12, 45, and 2.25 mm, respectively. The artery wall had a thickness of 0.5 mm and the artery was placed at about 4/5 of the soft tissue radius from the center. The bone was set as the fixed boundary, as its elasticity was more than 10 times greater than the other components. The soft tissue was assumed to be composed of linear elastic materials; as the strain was less than 10% during the blood pressure measurement and within the range it exhibits linear elastic behavior^23^. The soft tissue was modeled with an elastic modulus of 47.5 kPa and a Poisson’s ratio of 0.45^34^ and the artery wall was modeled with an elastic modulus of 450 kPa and a Poisson’s ratio of 0.45^23,30^. Here, the radius, elasticity, and thickness of the artery and the elasticity of the soft tissue were varied for the parametric study. The baseline values were 2.25 mm, 450 kPa, 0.50 mm, and 47.5 kPa, respectively, and the values were decreased and increased by 10% to examine the corresponding changes in the oscillometric signal.

**Figure 3:**
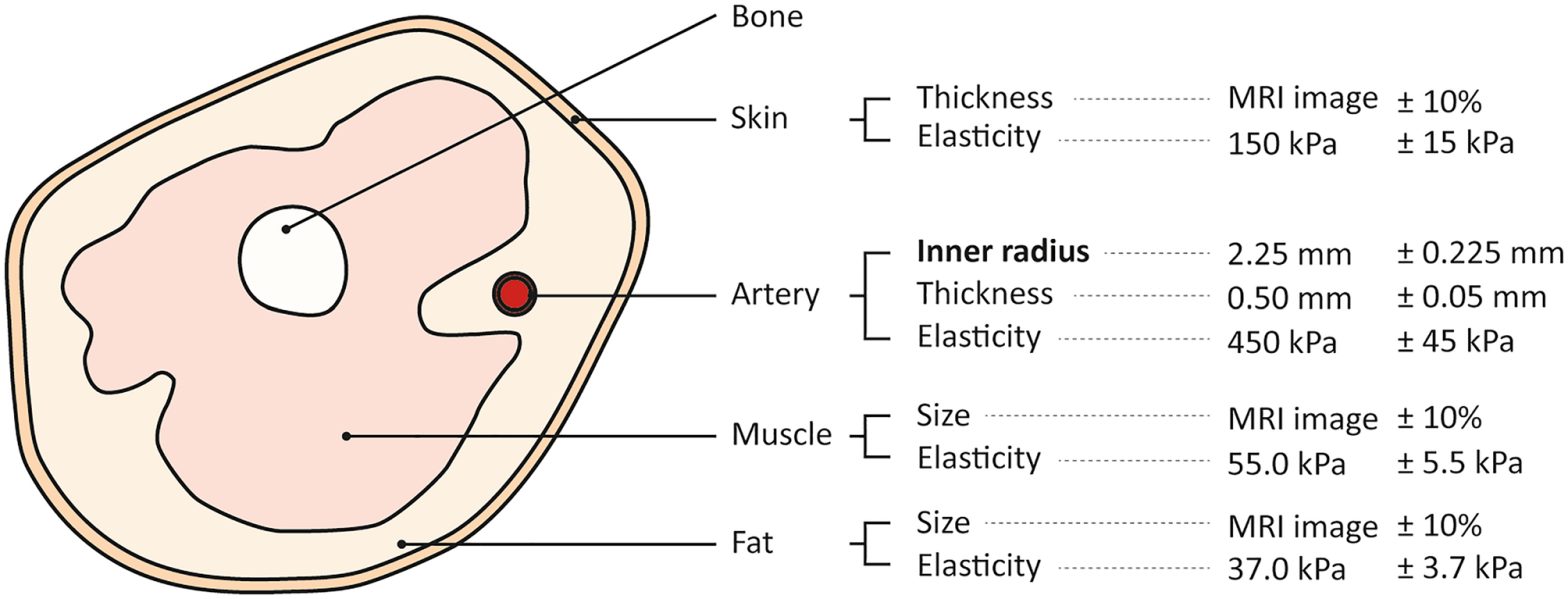
Upper arm parameters varied in the parametric study and experiment. Four physiological components were considered in the parametric study. Nine parameters in the four physiological components were investigated in the parametric study with the MRI-based FEM model, and among them, the inner radius of the artery (in bold) letters is varied within phantoms for the experiment. The baseline and the range of the parameters’ values used for the parametric study are presented.

For the MRI-based FEM model shown in Fig. 2d), the configuration was taken from an actual MRI image. The width and height of the entire model were 89.5 and 80 mm, respectively. As in the simple FEM model, the interface between the bone and the muscle was set as the fixed boundary. The muscles were modeled as one muscle layer surrounding the bone. The skin, fat, and muscle were assumed to behave as linear elastic material, since the strain was less than 10% during blood pressure measurement, and within this range they exhibit linear elastic behavior^23^. The skin was modeled with an elastic modulus of 150 kPa and a Poisson’s ratio of 0.45^36,37^. The fat was modeled with an elastic modulus of 37 kPa and a Poisson’s ratio of 0.45^34^. The muscle was modeled with an elastic modulus of 55 kPa and a Poisson’s ratio of 0.45^34^. Finally, the artery wall was modeled with an elastic modulus of 450 kPa and a Poisson’s ratio of 0.45^23,30^. For the MRI-based model, the nine upper arm parameters chosen for the parametric study were: the inner radius, thickness, and elasticity of the artery; thickness and elasticity of the skin; size and elasticity of the muscle; and, size and elasticity of the fat. The baseline values for the inner radius, thickness, and elasticity of the artery were 2.25 mm, 0.50 mm, and 450 kPa, respectively, and the elasticity of the skin, fat, and muscle were 150 kPa, 37 kPa, and 55 kPa, respectively. For the thickness of skin, and the size of muscle and fat, the baseline sizes were obtained directly from the MRI image, and the size was increased and also decreased by 10% from the baseline value proportionally. The baseline values are decreased and increased by 10%, and the corresponding changes in oscillometric signal were examined. The parameters used in the parametric study are schematized in Fig. 3.

### Experiment with cardiovascular simulator and phantoms

#### Phantom fabrication

In order to reproduce the actual blood pressure measurement process experimentally, an upper limb phantom was fabricated using silicone, as shown in Fig. 2e. The cross-section of the phantom was designed based on the same MRI image used in the FEM model, and the mold for the phantom was printed using a 3D printer based on the MRI image with a whole length of 18 cm. The phantom comprised the bone, muscle, fat, skin layer, and artery space. The artery was designed as a hollow space inside the fatty layer due to the complexity of forming silicone into a thin-wall tube like a brachial artery. The bone was made with the heat-resistant polyurethane resin (Task™ 8, Smooth-On, Inc.), and then the muscle-mimicking silicone rubber (Ecoflex™ 00–20, Smooth-On, Inc.) was poured around the bone encapsulate by the muscle mold. Following curing, the entity of the bone and muscle was put into the arm mold, and a circular rod with a radius of the brachial artery was placed at the brachial arterial position, which was to be removed once the silicone had cured. A fat-mimicking silicone rubber (Ecoflex™ 00–10, Smooth-On, Inc.) was then poured around and hardened. The skin layer was made separately, with silicone rubber (Dragon Skin™ 10NV, Smooth-On, Inc.). The cross-sectional size of the phantom was the same as the MRI-based FEM model, which was described in the Finite Element Method (FEM) Model subsection. With three different inner radii of 2.00, 2.50, and 2.75 mm, three phantoms were fabricated to verify the correlation between the inner radius of the artery and the oscillometric signal.

#### Cardiovascular simulator (virtual patient)

**Figure 4:**
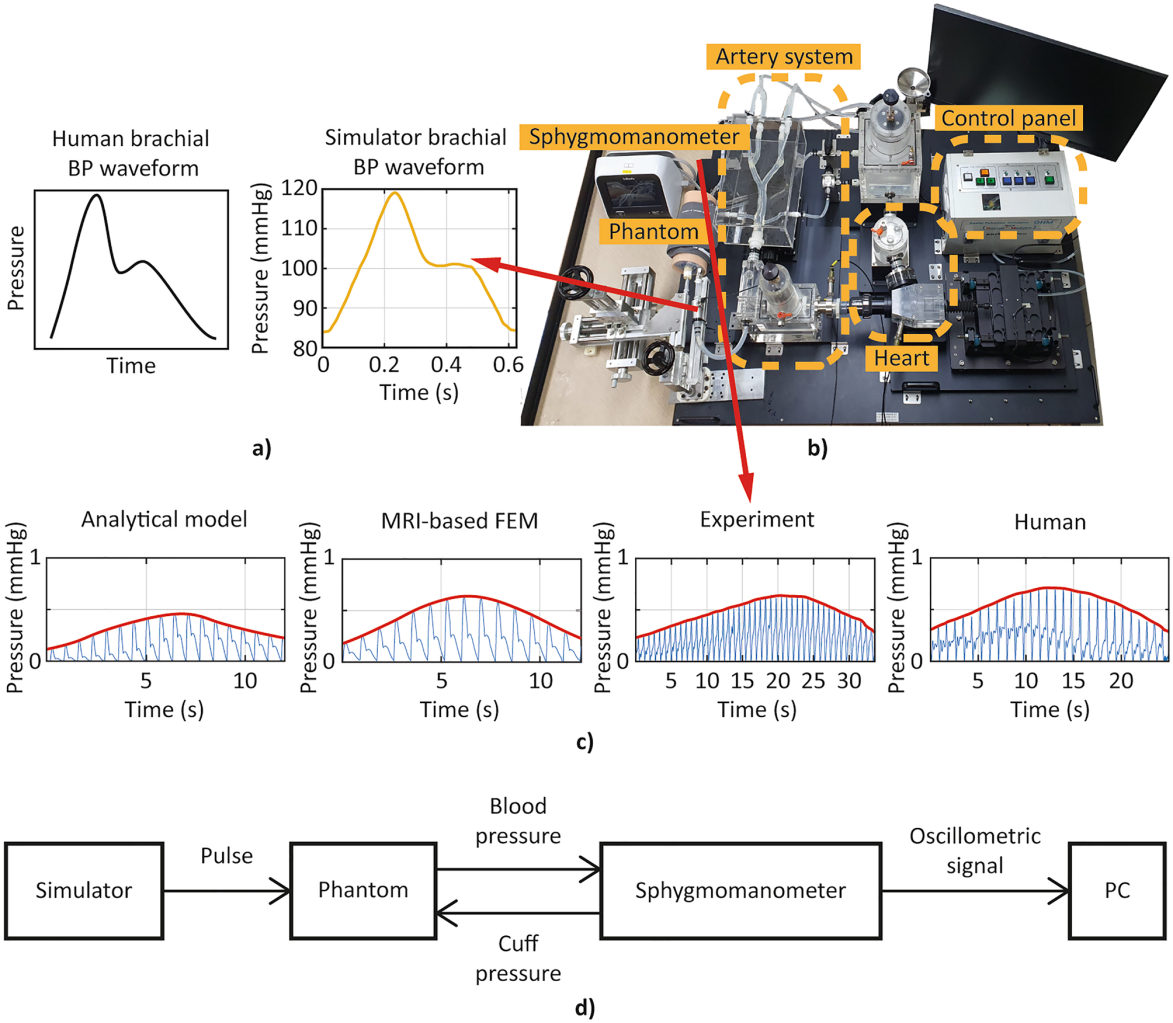
Experimental setup and flow chart with the cardiovascular simulator and the signals obtained from the experiment. (**a**) Human brachial pulse waveform and that created by the cardiovascular simulator, recorded just before the phantom, (**b**) Experimental setup with the cardiovascular simulator: pump mimicking heart is actuated to create the pressure waveform that reproduces the pulse wave in the human circulatory system, (**c**) Oscillometric signals obtained from the analytical model, MRI-based FEM model, experiment, and human, respectively. (**d**) Flow chart of the experimental procedure.

The cardiovascular simulator^38^ shown in Fig. 4b was utilized to create the appropriate pressure waveform that mimics the human blood pressure. The simulator comprised the pump (the *heart*), where it can make various input waveforms to the system, and the tubing system (the *artery system*), where the material and the formation are similar to the human circulatory system. An example of a brachial blood pressure waveform created by the cardiovascular simulator is shown in Fig. 4a, with actual human brachial blood pressure waveform for comparison.

#### Experimental setup

The brachial blood pressure waveform was reproduced from the cardiovascular simulator, and the phantom was installed in the upper arm position in the simulator. A sphygmomanometer cuff was put around the phantom to replicate a standard blood pressure test on a real arm and the corresponding oscillometric signals were collected through the sphygmomanometer. The pressure wave right before the phantom was monitored through the whole experiment (Fig. 4a). Figure 4c showed example oscillometric signals obtained from the analytical model, FEM model, experiment, and human subject, respectively, and Fig. 4d shows the overall experiment process.

### Evaluation index

#### Meaningful range

**Figure 5:**
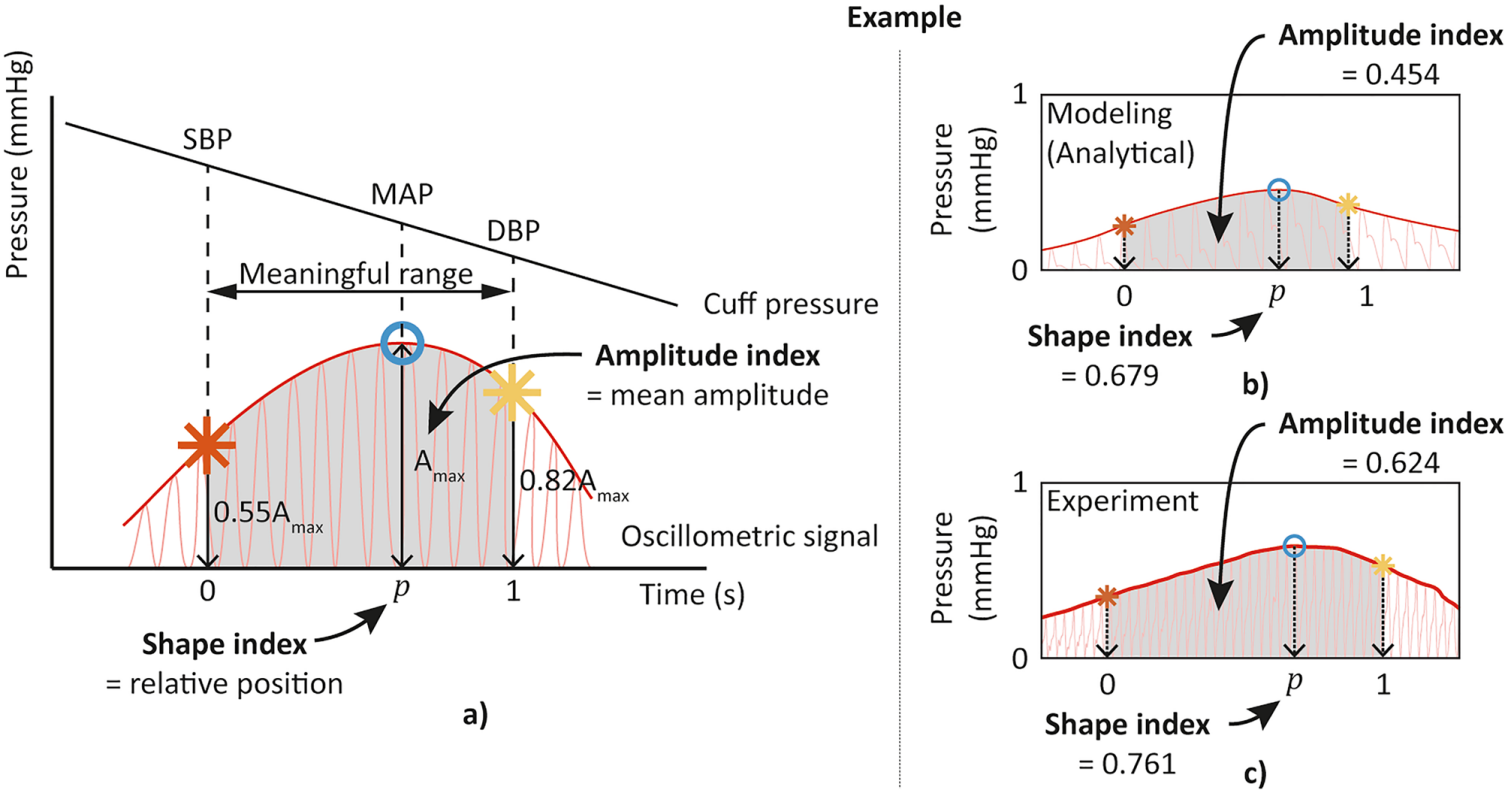
Oscillometric signal evaluation indices with meaningful range indicated. (**a**) Setting of the meaningful range of the oscillometric signal based on the maximum amplitude algorithm, and definition of an amplitude index and a shape index. Example calculation of two indices with the oscillometric signal obtained through (**b**) the modeling, and (**c**) the experiment.

Unlike the signals obtained from the simulation with modeling, each oscillometric signal obtained from the experiment had a different time length depending on the maximum pressure the device inflated. Hence, in this work, a “meaningful range” was set (Fig. 5a), where evaluating indices were defined to compare the signals from the commercial BP device without undesired bias. The meaningful range was set according to the maximum amplitude algorithm^33^ (MAA), which is the most generally used algorithm for commercial automatic sphygmomanometers. In the MAA, the systolic blood pressure (SBP) and diastolic blood pressure (DBP) were estimated to be the cuff pressures that matched to the amplitudes of certain ratios to the maximum amplitude of the oscillometric signal. According to Geddes^33^, the most generic ratios are 0.55 before maximum for SBP and 0.82 after maximum for DBP. Therefore, the point where the amplitude is 0.55 of the maximum amplitude before maximum value, and the point where the amplitude is 0.82 of the maximum amplitude after the maximum are set as each end of the meaningful range. From here, the amplitude and the shape of the oscillometric signal could be evaluated.

#### Amplitude index

The amplitude of the oscillometric signal was evaluated by the mean amplitude of the signal envelop within the meaningful range, as shown in Fig. 5a.

#### Shape index

The shape of the oscillometric signal was compared by the relative position *p* of the maximum amplitude within the meaningful range, as shown in Fig. 5a. The relative position was calculated by setting each end of the meaningful range as 0 and 1, respectively.

Figure 5b and c show the example calculations of both indices with oscillometric signals obtained through the modeling and the experiment, respectively. The meaningful range (gray), amplitude index, and shape index are indicated in each case.

## Results

**Figure 6:**
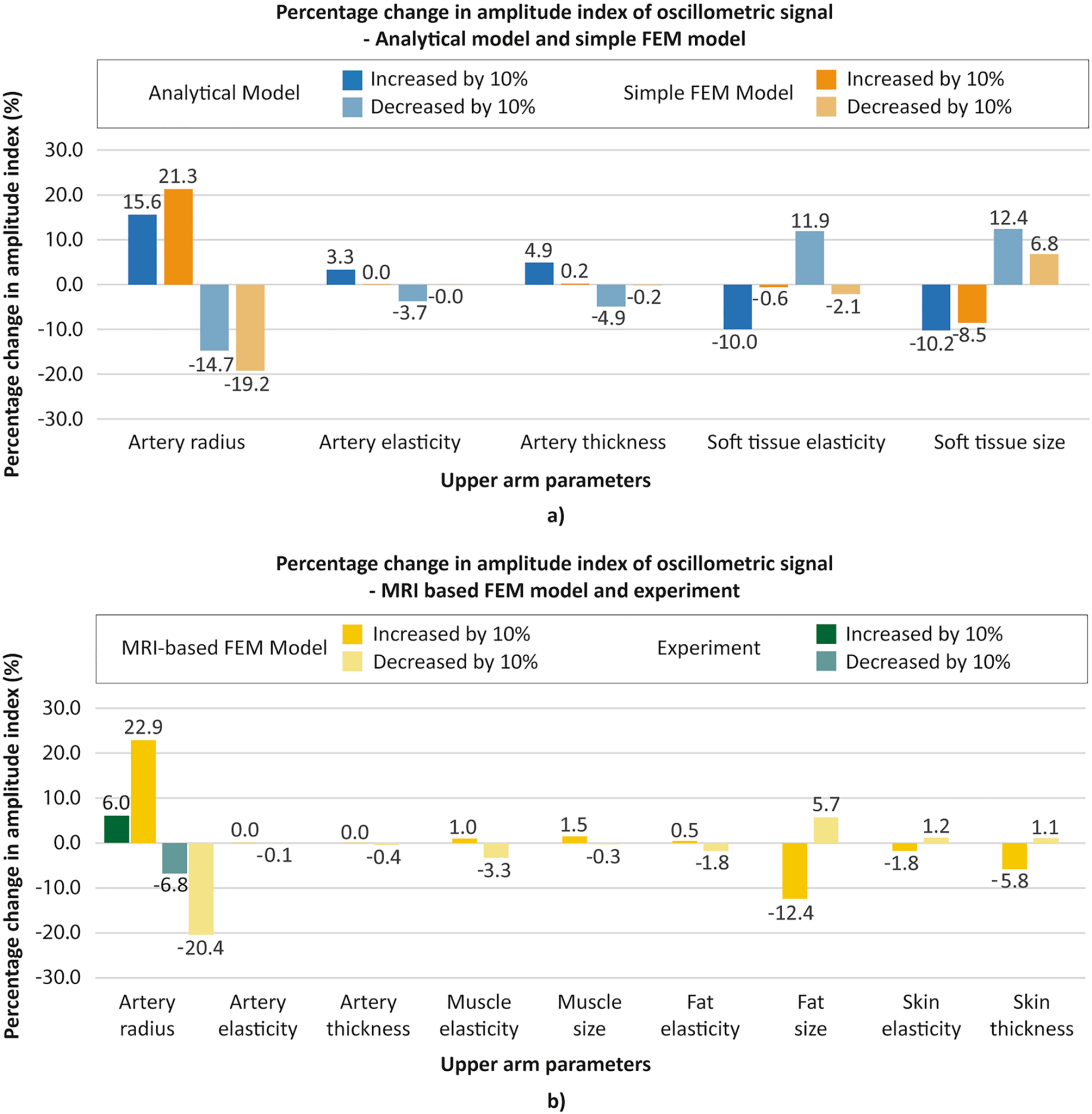
Parametric study results. The percentage change in amplitude index of oscillometric signal when upper arm parameters are varied for each model. (**a**) Parametric study results with analytical model and simple FEM model, (**b**) Parametric study results with MRI-based FEM model and experiment.

The correlation analysis results based on the amplitude index with the analytical model, the simple FEM model, the MRI-based FEM model, and the experimental phantoms in the cardiovascular simulator are shown in Fig. 6. From all three models (analytical, simple FEM, and MRI-based FEM model, indicated in blue, orange, and yellow, respectively), it was revealed that among all of the selected parameters, the variation of radius of the artery has a dominant effect on the change of the oscillometric signal amplitude. The artery radius is positively correlated with the amplitude of the oscillometric signal, implying that the amplitude of the oscillometric signal decreases as the blood vessel narrows. The consistent results are also drawn from the experiments with the phantoms.

In Fig. 6a, the parametric study results from the analytical model and the simple FEM model are presented. Comparing the two results from the models, the artery radius becomes more influential in the simple FEM model compared with the analytical model. Moreover, the result shows that soft tissue size inversely affects the amplitude of the oscillometric signal, implying that as the arm size becomes larger, the oscillometric signal recorded becomes smaller.

Figure 6b shows the correlation analysis of more upper arm parameters. As in the analytical and simple FEM results, the MRI-based FEM result shows that the artery radius has the most dominant effect on the amplitude of the oscillometric signal. Among the newly added parameters, the size of the fat affects the amplitude of the signal the most. This further clarifies that among the soft tissues (muscle, fat, and skin), whose size shows a considerable effect on the oscillometric signal amplitude based on the simple FEM model in Fig. 6a, that fat accounts for the effect the most. The experiment results show a consistent trend in that as the inner radius of the blood vessel becomes larger (smaller), the amplitude of the signal becomes larger (smaller). Here, the experiment results show a much smaller effect compared with the modeling results. This phenomenon can be explained by decreased flow, and hence, less force, due to the vessel network in the cardiovascular simulator used in the experiment. The simulator has an artery system that shares the flow, thus reducing the flow of the brachial artery when the cuff exerts pressure on the arm, whereas modeling does not consider the effect of neighboring vessels.

**Figure 7:**
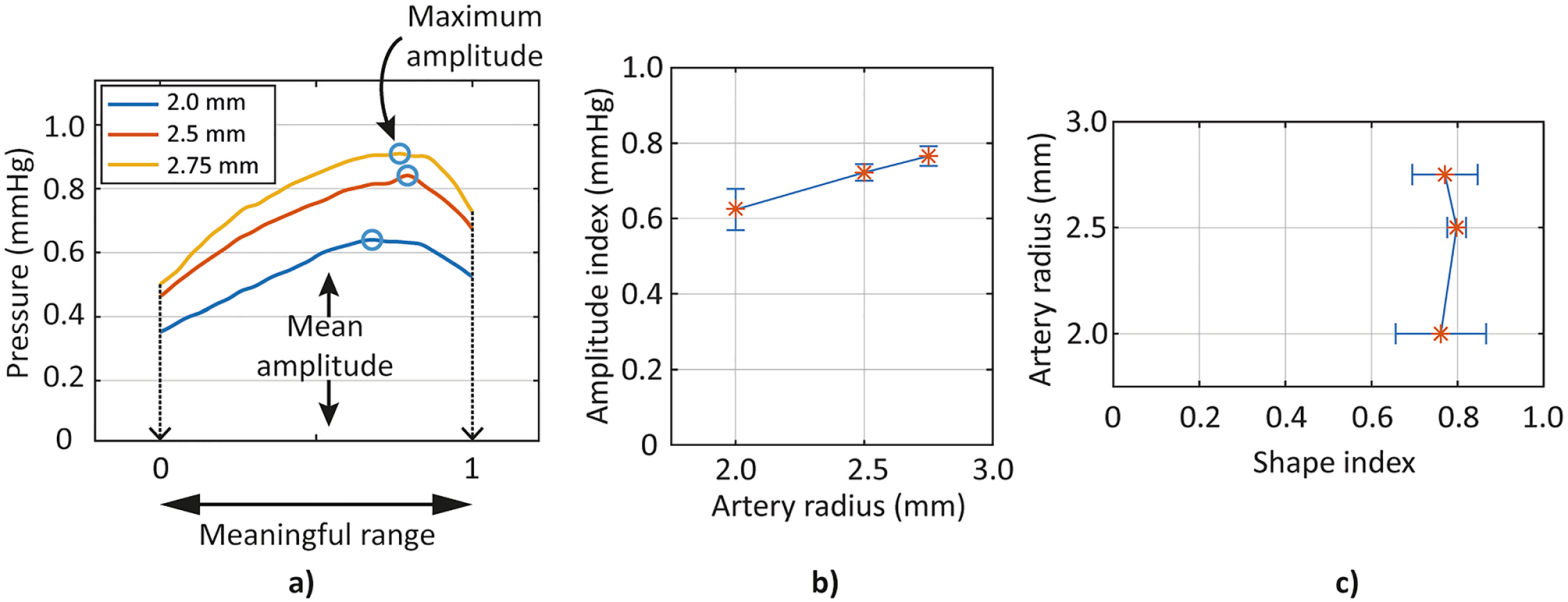
Correlation analysis between inner artery radius and oscillometric signal—Experiment with phantoms. (**a**) Oscillometric signals (envelope only, time-axis normalized to the meaningful range) obtained from the experiment with different artery radius, (**b**) Amplitude index (ordinate) with various artery radius, (**c**) Shape index (abscissa) with various artery radius.

The correlation analysis results based on the shape index show no clear trends in that the changes in the upper arm parameters do not trigger change in the shape index of the oscillometric signal. In Fig. 7, the correlation analysis between the inner radius of the artery and the oscillometric signal based on the experiment results (Fig. 7a) are shown. As already indicated in Fig. 6, the signal amplitude shows clear tendency with the artery radius (Fig. 7b), whereas the shape of the signal does not show a particular tendency with the artery radius (Fig. 7c). Since the shape of the oscillometric signal is utilized to estimate the blood pressure in commercial sphygmomanometers, the result that the position of the maximum signal has no relevance to the upper arm parameters is reasonable, considering that the input pressure level exerting on the inner wall of the artery is kept unchanged throughout the entire modeling simulation and experiment process.

## Discussion

The main result obtained from the analytical/FEM modeling and the experiment is that the artery radius affects the amplitude of the oscillometric signal the most, and the shape of the oscillometric signal is relatively impervious to the variation of upper arm parameters. As seen in Eqs. (2) and (3), if the inner radius of the artery increases by 10%, the change in radial displacement increases by similar order. Compared to the MRI-based FEM modeling result, the experiment results show a smaller effect on the amplitude of the oscillometric signals. This can be explained by the following reasons: First, the upper arm components are modeled as linear elastic materials. However, the silicone used for the muscle, fat, and skin are usually modeled as non-linear, hyper elastic materials, which may show different responses under the pressures exerted by the blood pulse and the cuff. Moreover, the experimental setup has a vessel network similar to a human body, where the blood vessels are connected like a web. This may reduce the effect from the changes in the artery radius. When the cuff exerts pressure on the phantom, the brachial artery resistance increases and the flow may go through other neighboring vessels. Since the FEM modeling does not consider the existence of the neighboring vessels, the percentage change in the FEM modeling and the experiments may show different results, that is, the experiment shows a smaller change of oscillometric signal amplitude. Nevertheless, both results suggest that the amplitude of the oscillometric signals is closely related to the change in blood vessel radius.

The shape of the oscillometric signal shows no noticeable trends with the upper arm parameters; the shape index showed insignificant change (less than 3.5% change in shape index with 10% change of parameters) with no specific tendency with increasing (decreasing) parameters, like the case described in Fig. 7c. As illustrated in Fig. 5a, the fundamental algorithm of estimating blood pressure using an oscillometric signal is to use the envelope of the oscillometric signal, reading the cuff pressure when the envelope of the signal becomes a certain portion of the maximum amplitude of the envelope. With the fixed deflation rate of the cuff pressure and the blood pressure (120/80 mmHg) inside the brachial artery during the parametric studies, the insignificant changes in the shape of the oscillometric signal support the validity of the MAA algorithm that can estimate the blood pressure regardless of the change of upper arm parameters.

Considering the case in which a person measures their blood pressure at rest every time but that the amplitude of the oscillometric signals tend to decrease as times goes by, then this can alert the person to the possibility of a narrowed vessel. Although the change in the artery radius might not be significant, the long-term trend may help to predict any change in the artery radius such as atherosclerosis. It will be appropriate if the person measures their blood pressure regularly for a certain duration of time and store the data, and any trend in the amplitude of the oscillometric signal may give extra information about the artery radius other than blood pressure alone. The inner radius of the artery is important, since it is closely related to CVDs. In fact, atherosclerosis, which results in narrowed blood vessel, is one of the most common factors that causes complications such as coronary artery diseases, carotid artery diseases, or aneurysms, depending on which arteries are blocked. Therefore, regular blood vessel checks will help to detect diseases caused by blood vessel deformation before they have progressed too far.

In order to have more clinically meaningful implications, further studies should be conducted. Notably, this work considered brachial artery parameters only. However, degenerative changes in blood vessels are not limited to the brachial artery; atherosclerosis can occur anywhere where blood vessels exist, including in coronary arteries, the aorta, and peripheral arteries. Thus, studies regarding the location where vessel deformation occurs the most should be conducted, and whether parameter changes in other arteries other than the brachial artery can be detected from oscillometric signals obtained at the upper arm should be studied. Indeed, parameters related to larger arteries like the aorta have a more crucial meaning clinically. Therefore, further research should be conducted with the cardiovascular simulator introduced in Fig. 4b to identify the correlation between the oscillometric signal and the cardiovascular parameters, not just constrained to the upper arm but with the aorta and other major arteries.

Nevertheless, the brachial artery diameter itself possesses clinical importance, too. The relationships between the brachial artery diameter and cardiovascular risk such as age, body mass index, systolic blood pressure, diastolic blood pressure, triglyceride level, high-density lipoprotein (HDL) cholesterol level, glucose level, as well as Framingham risk score^39,40^ have been reported, suggesting the brachial artery diameter as a potential tool to predict cardiovascular events. Also, previous studies regarding the correlation between the brachial artery diameter and right ventricle (RV) mass, RV end-diastolic volume (RVEDV), RV ejection fraction (RVEF)^41^, and coronary artery disease^42^ showed the clinical relevance of the brachial artery diameter with cardiovascular events. Considering that the brachial artery diameter is assessed by ultrasonography in current clinical practice, extracting the brachial artery radius from an automatic sphygmomanometer will be helpful for the early diagnosis and treatment of cardiovascular events reported in the previous works^39–42^.

In summary, this work shows the correlation between the inner radius of the brachial artery and the oscillometric signal, suggesting the possibility of utilizing oscillometric signal to monitor the brachial artery diameter. This work is the first step to further research and develop the use of oscillometric signals as a cardiovascular health monitoring index.

## Conclusion

In this work, the correlation between upper arm parameters and oscillometric signals were studied. In order to analyze the correlation, parametric studies based on analytical and numerical modeling were conducted. The parametric study results showed that the inner radius of the artery has the most influence on the amplitude of the oscillometric signal among the upper arm parameters, whereas the shape of the oscillometric signal shows no significant changes with different upper arm parameters. After the parametric studies with modeling, upper arm phantoms with various artery inner radii were fabricated in order to verify the effect of the parameters on the amplitude of the actual oscillometric signal through the experiments. The experimental result shows that the radius of the artery affects the amplitude of the oscillometric signal the most, giving an average of 6.5% change in the amplitude when the radius is changed by 10%, while the shape of the oscillometric signal obtained from the experiments shows trivial changes. Throughout the modeling and the experiments, the correlation between the amplitude of the oscillometric signal and the artery parameters are shown and the results demonstrate the possibility that the oscillometric signal can be used as a monitor of brachial artery diameter.

## Data Availability

The datasets generated during and/or analyzed during the current study are available from the corresponding author upon reasonable request.
